# Maintaining independence at home after a fall: a process evaluation of the MAINTAIN multicomponent intervention for people living with dementia

**DOI:** 10.1093/ageing/afaf245

**Published:** 2025-09-10

**Authors:** Leanne Greene, Louise M Allan, Alison Bingham, Ashima Sharma, Bethany Whale, Robert Barber, Christopher Fox, Victoria A Goodwin, Adam Lee Gordon, Abigail J Hall, Rowan H Harwood, Claire Hulme, Thomas Andrew Jackson, Rachel Litherland, Steve W Parry, Obi Ukoumunne, Sarah Morgan-Trimmer

**Affiliations:** University of Exeter Medical School, CTU, College House St. Luke’s Campus, Exeter, Devon EX1 2LT, UK; University of Exeter Medical School, Exeter, Devon, UK; University of Exeter Medical School, Exeter, Devon, UK; University of Exeter Medical School, Exeter, Devon, UK; University of Exeter—Clinical Trials Unit, Exeter, UK; Cumbria Northumberland Tyne and Wear NHS Foundation Trust—Campus for Ageing and Vitality, Newcastle upon Tyne, UK; University of Exeter Medical School, Exeter, Devon, UK; University of Exeter Medical School, Exeter, Devon, UK; University of Exeter Medical School, Exeter, Devon, UK; Queen Mary University of London—Wolfson Institute of Population Health, London, UK; Barts Health NHS Trust—Academic Centre for Healthy Ageing, London, UK; University of Exeter Medical School, Exeter, Devon, UK; University of Nottingham—School of Health Sciences, Queen’s Medical Centre, Nottingham NG7 2HA, UK; University of Exeter Medical School, Exeter, Devon, UK; University of Birmingham—Institute of Inflammation and Ageing, Centre for Translational Inflammation Research, Queen Elizabeth Hospital, Mindelsohn Way, Birmingham B15 2WD, UK; Innovations in Dementia, Exeter, UK; Newcastle Upon Tyne Hospitals NHS Foundation Trust—Falls and Syncope Service, Newcastle upon Tyne, UK; University of Exeter—NIHR ARC South West Peninsula, Exeter, UK; University of Exeter Medical School, Exeter, Devon, UK

**Keywords:** falls prevention, process evaluation, older people, dementia, activities of daily living

## Abstract

**Background:**

People with dementia who have a fall can experience both physical and psychological effects, often leading to diminished independence. Falls impose economic costs on the healthcare system. Despite elevated fall risks in dementia populations, evidence supporting effective home-based interventions remains limited.

**Methods:**

Multiple-methods process evaluation within a pilot cluster randomised controlled trial informed by a realist approach. Settings included six UK sites/clusters (three intervention, three control). Fidelity checks on routine data collection and fidelity observations of intervention sessions, multidisciplinary team meetings and supervision sessions were undertaken. Semi-structured interviews were conducted with people with dementia, caregivers and intervention therapists.

**Results:**

The MAINTAIN intervention demonstrated high fidelity in home assessments and intervention delivery, with participants receiving a mean of 15 of the 22 available sessions with a range of 5–25 sessions. Qualitative findings revealed that regular home visits increased engagement and motivation. Multidisciplinary team support enhanced therapists’ confidence, particularly with complex cases. While most participants achieved their functional goals and reported improved confidence, challenges included geographical disparities in service delivery, carer burden and varying effectiveness of referral pathways. Therapists’ attitudes towards advanced dementia influenced intervention delivery. The paired approach, involving both the person living with dementia and their carer, supported activity engagement but occasionally added extra responsibilities for caregivers.

**Conclusions:**

MAINTAIN was both feasible and acceptable. Future studies should consider standardising multidisciplinary support, incorporating targeted falls-related anxiety support and establishing sustainable post-intervention maintenance strategies. Protocol adaptations, such as video consultations, showed promise in addressing workforce constraints.

## Key Points

- MAINTAIN, a home-based intervention for people with dementia who have had a fall, demonstrated feasibility, acceptability and high implementation fidelity while accommodating local resource variations.
- The process evaluation largely validated initial programme theories around multidisciplinary team (MDT) support, home visits and tailored care, while revealing implementation challenges including geographical accessibility, staffing constraints and the need for sustained post-intervention support.
- Future studies should focus on standardising MDT support, developing strategies to maintain therapeutic gains, targeted falls-related anxiety support, improving support for adult-child caregivers and establishing more effective referral pathways while considering alternative outcome measures to Goal Attainment Scaling.

## Introduction

In the UK, falls cost the healthcare system an estimated £4.4 billion annually [1]. People with dementia are at higher risk of falls than the general population [2, 3] and often experience poorer recovery outcomes [4], leading to reduced independence [5]. The impact extends beyond physical injury to include psychosocial consequences such as fear of falling and reduced confidence [6, 7]. There are ~982 000 people with dementia in the UK [8], with ~60% living in the community [9]. Despite the rising prevalence of dementia [10] and increased fall risk [2, 3], evidence supporting effective interventions remains limited [10].

Fall prevention interventions show mixed effectiveness in dementia populations—some improve physical function, but outcomes remain inconsistent [11–14]. National Institute for Health and Care Excellence (NICE) guidelines currently offer no specific recommendations for falls prevention in this group [15, 16]. Building on the DIFRID study (Developing an Intervention for Fall-Related Injuries in Dementia) [17], we developed MAINTAIN, a pilot cluster randomised controlled trial with an embedded process evaluation to explore implementation and refine intervention mechanisms for a future full-scale trial [18–20].

## Programme theory

**Figure 1:**
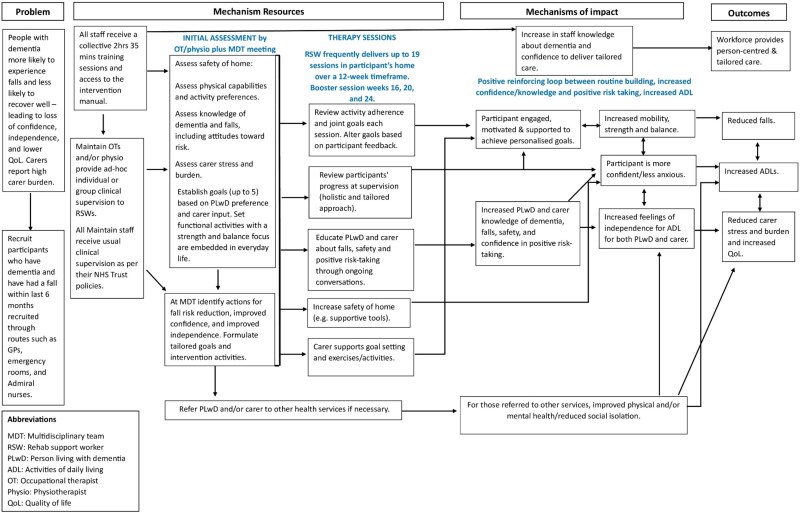
The original MAINTAIN logic model depicting the initial programme theory.

The programme theory, illustrated in a logic model (Fig. 1) and as if–then–because statements (Appendix 1), was developed from three sources: findings from DIFRID [17], stakeholder consultations and a review of dementia falls prevention literature. A detailed description of the intervention is published elsewhere [21] but Table 1 outlines the core components delivered for the MAINTAIN intervention. Our realist approach examined how the programme theory functioned in practice, focusing on (1) intervention fidelity, (2) contextual factors influencing engagement and (3) the extent to which observed mechanisms aligned with theoretical assumptions.

## Methods

### Design

We implemented the recommendations provided by the Medical Research Council to conduct this process evaluation [22]. We used a multiple-methods design, facilitating a deductive examination of *a priori* causal assumptions alongside an inductive exploration of participants’ and therapists’ experiences of the intervention. The process evaluation was conducted from October 2023 to December 2024. Approval for this study was obtained from the Wales REC 6 (Ethics reference 23/WA/0126); registered protocol ISRCTN16413728. The protocol for MAINTAIN has been published elsewhere [21]. We adhered to the consolidated criteria for reporting qualitative research (COREQ) [23] (Appendix 2).

### Settings and participants

Six sites/clusters were randomised, three to the intervention arm and three to the treatment-as-usual arm, although one intervention site and one control site failed to recruit participants. All eligible dyads (people with dementia and caregivers) at intervention sites, delivering therapists (physiotherapists, occupational therapists (OTs), rehabilitation support workers (RSWs)) and clinical researchers were invited to participate in the process evaluation. Of the 18 participant–carer dyads enrolled in the intervention arm, 10 participated in an interview. In addition, one service manager, six clinical researchers involved in outcome data collection, and seven intervention staff (including physiotherapists, OTs and RSWs) volunteered to participate in interviews. Site characteristics and participant demographics are provided in Appendices 3 and 4, while full trial recruitment and progression details will be reported in the main trial publication.

### Data collection

Data collection comprised observations (two in-person and four audio-recorded therapy sessions, two virtual multidisciplinary team (MDT) meetings and one supervision session), using a checklist to capture qualitative and quantitative fidelity data (Appendix 5). Case report form data provided additional fidelity measures. Semi-structured interviews (Appendix 6) were conducted post-intervention with people with dementia and their caregivers, therapists and clinical researchers. Of 24 interviews, 13 were conducted via Microsoft Teams (12 professional, 1 dyad) and 11 via telephone (1 professional, 10 dyads). An experienced qualitative researcher (L.G.), independent of intervention design and implementation, conducted all interviews and observations. Trial outcomes will be reported in a subsequent publication.

**Table 1:** Core components delivered for the MAINTAIN intervention

**Intervention components: mechanism resources and delivery framework**
Workforce set-up (training) and delivery (multidisciplinary team working and supervision)
Frequent face-to-face home visits
Holistic assessment and care
Tailored care and goal-setting
Consistent person-centred care
Functional activities with a strength and balance focus are embedded in everyday life
Education on falls
Caregivers support and prompt the intervention between intervention sessions

**Table 2:** Fidelity checks using case report form data for home assessments (*n* = 17)

**Variable**	**Finding**
Occupational therapist present	17 (100%)
Physiotherapist present	7 (41%)
Carer present	17 (100%)
Generic assessment completed	17 (100%)
General observations and posture checked	17 (100%)
Timed up and go assessment completed	7 (41%)
Home environment checked	12 (70%)
Self-care/productivity assessed	16 (94%)
Affect and self-awareness assessed	17 (100%)
Cognition assessed	17 (100%)
Perception and sensory impairments assessed	15 (88%)
Actions assessed	15 (88%)
MDT meeting logged on REDCap database	1 (6%)
Problems logged and referrals made	8 (47%)
Goal Attainment Scaling (GAS) conducted	16% (94%)

Note: While there is no universal consensus on optimal adherence levels, implementation is generally considered high fidelity at 80%–100% and low fidelity at ≤50% [24–26].

### Data analysis

#### Quantitative

Intervention adherence and observational fidelity checklist data were summarised using descriptive statistics using Microsoft Excel [27].

#### Qualitative analysis

To gain a detailed understanding of causal processes and refine the programme theory, a realist-informed approach was used to analyse how various aspects of delivery activated mechanisms and influenced changes in both people living with dementia and their caregivers, contributing to the understanding of the observed outcomes. Interviews were anonymised and transcribed verbatim for analysis in NVivo v.14. Analysis followed Jackson and Kolla’s [28] realist approach, using Dalkin’s [29] conceptualisation of mechanisms as resources and responses (detailed in Appendix 7).

### Quantitative results

The MAINTAIN training was delivered to all therapists in the intervention arm. Home assessment fidelity to the protocol was generally high Table 2), although at Site 1, physiotherapy capacity constraints limited initial assessments. This was not a protocol deviation as MAINTAIN allowed either OT or physiotherapist completion, with video consultations implemented as an adaptation. However, capacity limitations affected ‘Timed Up and Go’ test administration and potentially influenced other assessment components. Although MDT meetings were not formally documented, evidence from team discussions and interviews confirmed that these meetings were routinely taking place.

**Table 3:** MAINTAIN observation sessions using the MAINTAIN fidelity checklist tool

**Observation**	**Score**	**Percentage**
Site 1 MDT	18/24	75%
Site 1 supervision	12/14	86%
Site 1 intervention session	32/44	73%
Site 1 review session	32/42	76%
Site 2 MDT	6/24	25%
Site 2 intervention session	37/44	84%
Site 2 intervention session	38/44	86%
Site 2 intervention session	35/44	80%
Site 2 intervention session	40/44	91%
		**Mean percentage for observations:** 75%

Note: Site 2 did not undertake supervision.

Intervention sessions showed moderate adherence to the manual**:** 69% of dyads (11/16) received all sessions from the same RSW. Participants completed a mean of 15 out of a possible 22 sessions (SD = 0.007; range: 6–25), meeting the predefined stop/go criterion of attending at least 14 sessions (≥60%). Sessions lasted an average of 62 min (SD = 0.01; range: 10 min to 2 h 45 min), aligning with the protocol-defined target of 60-min sessions.

Caregivers were present at 175 out of 207 sessions (85%), indicating strong family engagement. Data on whether caregivers were present were missing for 37 sessions. Observational data using fidelity checklists (Appendix 4) confirmed moderate implementation fidelity across intervention delivery, MDT discussions and supervision components (Table 3).

### Qualitative results

**Figure 2:**
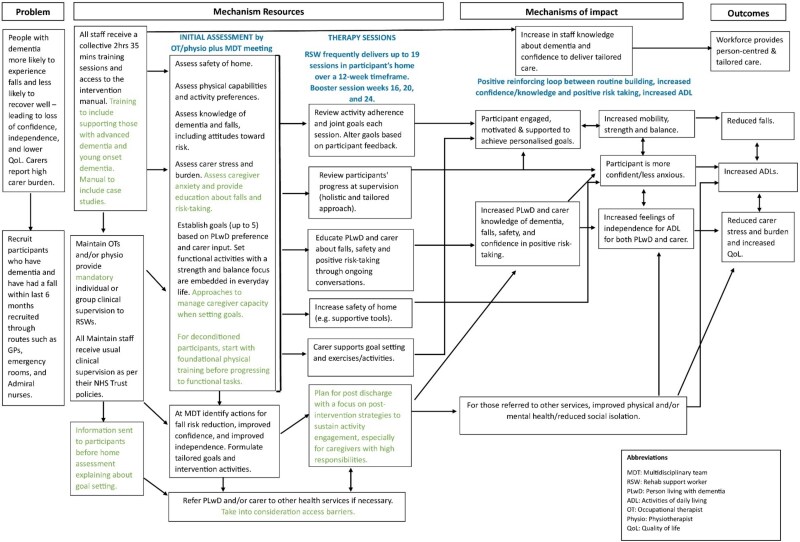
The refined MAINTAIN logic model.

To maintain confidentiality with our small sample, quotations are presented without site or role identifiers, using pseudonyms. The revised programme theory is presented in an amended logic model (Fig. 2), and additional findings are presented in Appendix 10.

### MDT, supervision and training

The programme theory around MDT support enhancing tailored care delivery was affirmed. Therapists demonstrated greater capability and confidence when robust MDT structures, supervision and strong clinical leadership were in place.

*‘the MDT’s been really good to help each other… just to have that debrief and support each other… also an opportunity for us to discuss what can we change? What can we adapt?’* (Therapist)

MDT support was particularly important when participants had complex unmet needs. Conversely, in settings with limited MDT support, especially for support staff, feelings of isolation and vulnerability were more prevalent.

*‘As an RSW, you're not qualified, you can't make decisions… when you're asking for help and it's not really being given… it just doesn't feel balanced at all.’* (Therapist)

Our initial programme theory proposed that structured training would enhance therapists’ clinical competencies and person-centred dementia care. However, most therapists already had dementia care experience and felt the MAINTAIN training added little new knowledge. An unanticipated factor was therapists’ attitudes towards advanced dementia, which shaped how they understood meaningful outcomes. Some were sceptical about the intervention’s effectiveness for individuals with more severe dementia.

‘*who gets accepted onto the project – needs to be… I think they need to have a higher cognitive ability… realistically, whether or not the goals change, I'm not convinced, because they started off in such a low state.’* (Therapist)

Others recognised the clinical significance of incremental gains.

‘*I mean, obviously the people at those early stages, I would say the benefits will be more but, you know, even for the people with more advanced dementia, some of those little gains are huge.’* (Therapist)

### Tailored care and goal-setting

Our programme theory postulated that tailored care with individualised goals would make activities more meaningful and achievable. There was evidence that this was supported.

*‘I told them about strength in her legs, standing, and wanting, you know, the help, because it would help me… things are tailored round that.’* (Caregiver)

However, complex care needs and dementia symptoms—especially lack of insight—affected the effectiveness of tailored care. Caregivers felt therapists needed to understand both the dyad’s dynamics and dementia symptoms to set meaningful, achievable goals. One carer perceived a lack of such tailoring.

*‘It [tailored goal setting] shows a sort of lack of understanding of where that particular patient is… there are different things for each person… I think they were finding us quite difficult beside the requirements of the achievable goals.’* (Caregiver)

Therapists who were more experienced in dementia care showed a greater ability to adapt and tailor their therapy sessions to meet individual needs.

*‘So she'd come in with a plan, and then sometimes they'd deviate from the plan and kind of do it a different way that kind of fitted in with Steve.’* (Caregiver)

In comparison, therapists who specialised in general rehabilitation tended to focus on a more goal-orientated approach.

‘*I think she knew her programme of exercises… and whether Bex wanted her to push her knee or not, she was going to push it, because that was the programme of exercises.’* (Carer)

In terms of setting tailored goals, all therapists reported that the GAS was time consuming, evidenced by its low completion rate in the fidelity data, as it often required post-home assessment session completion. Therapists also felt that the GAS was not conducive to establishing functional goals in dementia care contexts.

*‘in this clientele, I think the cognitive ability needs to be taken into account when they're setting goals, because it's really hard to get them to come up with a realistic goal in the first place. And that includes the carer.’* (Therapist)

Several participants felt they achieved the goals they set during MAINTAIN and they felt more confident and independent.

*‘And I wanted to be more independent, I really did, and I've gained that independence. And I wanted to be more confident, and, to a certain extent, I've got that confidence. So, I feel that MAINTAIN has done a great deal for me.’* (Person with dementia)

### Education on falls

We hypothesised that assessing participants’ knowledge and attitudes towards falls would enable more targeted education to support risk management. Findings supported this, particularly among dyads receiving fragmented care, where limited professional input coincided with worsening symptoms. Education improved confidence, knowledge and risk management behaviours, contributing to enhanced quality of life.

*‘this is the first time they've [family] really had input… they got given a slide sheet [prior to MAINTAIN intervention], but didn't get told how to use it… us being there, and them physically having access to support, you know, now hopefully we've got some better practices in place.’* (Therapist)

Some participants felt that education reduced the incidence of falls at home.

*‘it’s reduced the falls in that sense… because they’ve… been able to assess and give us both some advice.’* (Caregiver)

Some caregivers, worried about recurring falls, restricting activities for the person with dementia. Therapists promoted balanced risk management by helping caregivers reduce protective behaviours and gradually encourage independent movement. This approach combined practical strategies and contingency planning, enabling caregivers to manage risk effectively while fostering independence.

*‘whenever he stood up, the daughter would basically be asking him where he was going, and then would follow him… helping them to understand that actually it's going to be less stress for both of you… to walk to the end of the drive….’* (Therapist)

Three caregivers reported that enhanced knowledge helped them identify their over-protective tendencies, noting how they had previously arranged support for the person with dementia due to uncertainty about risk management.

*‘We’ve always found another person to watch over her, almost babysitting her. That’s probably the wrong way to have dealt with it, but… you know? But that’s my lack of education on the subject.’* (Caregiver)

Despite education about balanced risk-taking, some caregivers experienced increased anxiety when introducing functional activities and remained unable to let the person with dementia participate in tasks they perceived as risky.

*‘But only cold drinks, I’m not having her boiling kettles and things like that, not yet. I haven't got that confidence in her. I don't want her to do anything that's going to endanger her wellbeing.’* (Caregiver)

### Carer supporting and prompting MAINTAIN activities

Our programme theory proposed that enrolling people with dementia and their caregivers together would enable ongoing support, as caregivers could provide prompts and encouragement when memory difficulties or motivation affected activity engagement. We found that this was a key element of MAINTAIN.

*'Having a carer that's heavily involved is really beneficial… they're establishing that routine… particularly if the person living with dementia has got apathy and low motivation.'* (Therapist)

The dyadic approach, while intended to support engagement, sometimes created an additional burden for caregivers. One participant articulated this challenge, describing how the responsibility to remember, implement and monitor activities added to their caregiving duties, questioning whether this truly promoted independence or merely transferred more tasks to the caregiver.

*‘I mean, the other thing is that you want to sort of avoid making more work for the carer… you've got to remember it, and you've got to action it, and you've got to check it all the time, it becomes kind of a bit of a, well, who's doing this, me or you?’.* (Caregiver)

One caregiver explained how the multiple steps of engagement sometimes influenced their decision to prompt intervention activities, highlighting the importance of finding a sustainable balance that works for both members of the dyad.

*‘Rather than sort of disturb him and get him up, and then have to explain what he's got to do… yeah, it sort of felt easier just to leave him.’* (Caregiver)

While therapists recognised the importance of caregivers’ support, they also worried about placing extra demands on caregivers, especially those dealing with their own health issues.

*‘it then relies on the carer to keep prompting… which from a carer perspective, can just get absolutely exhausting… which also makes you worry about how he is coping and managing with the situation.’* (Therapist)

Adult-child caregivers, unlike spousal caregivers, typically had to balance additional work and family commitments, intensifying their caregiving load. Despite outreach attempts, interviews with two adult-child caregivers could not be completed—one declined due to work commitments limiting their availability for research activities, while another did not respond to multiple contact attempts.

## Discussion

This process evaluation tested the initial MAINTAIN programme theory and generated insights to inform a future full-scale trial. Findings above and in Appendix 10 largely supported the theory, though with important nuances and unexpected outcomes. Fidelity data showed strong adherence to core components, though a mean delivery of 15 out of 22 sessions suggests the need to explore optimal intervention intensity.

The effectiveness of MDT support in enabling tailored care aligned with previous research [30], though implementation varied by site. Inclusive MDT structures and clinical leadership supported confident delivery, while their absence posed challenges, particularly for RSWs. Structured training provided limited new knowledge for therapists with existing dementia experience, but attitudes towards advanced dementia emerged as a key factor influencing delivery. Some therapists were sceptical about effectiveness in severe dementia, while others valued incremental gains. Future research should explore therapists beliefs about therapeutic potential in advanced dementia [31–33], alongside ongoing skills development [32, 34–36].

Programme theory around frequent face-to-face delivery revealed benefits and contextual challenges. Regular home visits maintained engagement and offered structure, particularly for those with young-onset dementia [37]. However, delivery was constrained by staff capacity in remote areas, a widespread issue in the UK [16], and concerns about intervention dependency, especially for highly involved caregivers [38], echoing previous findings [39]. These findings suggest home-based delivery is effective but must be supported by solutions for geographical access and post-intervention sustainability.

The intervention’s holistic approach addressed a critical gap in post-diagnostic dementia support, providing coordinated care where fragmentation is common [40]. Participants reported improvements in physical function, independence and quality of life, especially when managing comorbidities. However, implementation revealed inter-site variation in referral pathways, and unintended consequences of signposting (e.g. financial barriers and age-based service restrictions) highlighted access inequities, particularly affecting younger people with dementia [12, 41].

Individualised goal-setting and tailored care emerged as key components, though their success depended on factors like therapist experience. Dementia-specialist staff adapted approaches more flexibly than general rehabilitation therapists, who tended to use more rigid goal frameworks. Some appeared to believe those with advanced dementia would not benefit, possibly reflecting limited exposure or systemic patterns of exclusion from rehabilitation services [42, 43]. Overly ambitious goals risked increasing caregiver burden, especially where participants had limited insight. Future implementation should incorporate structured processes to align aspirations with caregiver capacity.

Similar to other studies [44], the GAS presented challenges in dementia care, particularly in setting realistic baselines and measuring small gains. Input from our PPIE group emphasised the difficulty of realistic goal-setting. Future trials may benefit from adapting GAS or exploring alternative measures better suited to dementia rehabilitation.

Fidelity data showed 69% of dyads received all sessions from the same RSW, reinforcing the value of consistent, person-centred care, critical in countering fragmented service experiences [40]. Participants felt supported and motivated by sustained therapeutic relationships. This was echoed by our PPIE group, who emphasised that trust and continuity are essential in understanding complex needs. Therapists’ outsider role also helped avoid tension that sometimes arose when caregivers encouraged activity.

Consistent with prior work [39], integrating functional tasks into daily life was valued by participants. This helped disrupt cycles of inactivity and loss of independence driven by physical decline and caregiver concerns. The intervention’s graduated approach appeared to improve strength, ADLs and quality of life. However, advanced frailty limited impact for some, indicating the need to consider broader, non-physical outcomes in progressive dementia [39]. Foundational physical capacity may be a prerequisite for advancing to complex tasks, though even simple activities offered meaningful benefits.

Falls education improved caregivers’ risk management but revealed tension between safety and independence. Therapists supported graded risk-taking and contingency planning, yet some caregivers remained anxious despite education. This suggests fall prevention in dementia requires ongoing support beyond initial education [45, 46].

Caregivers attended 85% of sessions and played a vital role in sustaining engagement, particularly for participants with motivation or memory challenges [39, 47, 48]. However, the dyadic model also imposed additional responsibilities on caregivers, particularly adult children balancing work and family [38, 49]. These findings suggest future models should include flexible support strategies tailored to different caregiver circumstances.

### Strengths and limitations

This study used a theory-based approach to understand factors shaping the delivery and receipt of MAINTAIN [50]. It also highlighted how outcomes such as improved ADLs can act as mechanisms for further change, such as reduced caregiver burden, illustrating the layered causal pathways in complex interventions. While this interplay was not fully modelled, recognising these dynamics can inform future refinements to the logic model.

As a pilot study, fidelity assessment focused appropriately on feasibility and core component delivery; more detailed, component-level fidelity analysis will be pursued in the definitive trial. The evaluation sample included only dyads who completed the intervention, potentially introducing selection bias. Capturing the perspectives of non-completers through alternative methods will be important for refining recruitment and delivery.

## Conclusions

This process evaluation demonstrated that MAINTAIN is feasible and acceptable, with strong protocol fidelity maintained across sites despite differing workforce structures. The findings supported initial programme theories related to MDT support, home delivery, holistic assessment and caregiver involvement, while identifying key implementation challenges, including geographical access, staffing constraints and referral system limitations. Adaptive strategies, such as video consultations, showed potential to address workforce gaps.

Future refinement should focus on standardising MDT support, especially for support workers; developing strategies to sustain gains post-intervention; enhancing support for adult-child caregivers; and improving referral pathways. Replacing GAS with outcome measures better suited to dementia care should also be considered. While interview data suggest positive impacts on ADLs and quality of life, a definitive trial incorporating these refinements is needed to assess effectiveness and implementation in diverse care settings.

## Supplementary Material

aa-25-0692-File002_afaf245
