# Supplementary material for: Maintaining independence at home after a fall: a process evaluation of the MAINTAIN multicomponent intervention for people living with dementia
Source: Age Ageing. 2025 Sep 10;54(9):afaf245. doi: 10.1093/ageing/afaf245 (PMC12421879; doi:10.1093/ageing/afaf245)
Supplement: aa-25-0692-File002_afaf245 [file aa-25-0692-file002_afaf245.docx]

**Contents Page**

**Page 2:** Appendix 1 - If-then-because statements to outline initial programme theory

**Page 7:** Appendix 2 – COREQ

**Page 10:** Appendix 3 - Site characteristics

**Page 13:** Appendix 4 - Description of process evaluation sample

**Page 16:** Appendix 5 - Fidelity checklists

**Page 18:** Appendix 6 - Interview guides

**Page 23:** Appendix 7 - Example of CMMOC analysis

**Page 25:** Appendix 8 - CMMOC pattern identification

**Page 26:** Appendix 9 - Expanded description of qualitative data analysis

**Page 28:** Appendix 10 - Additional qualitative results

**Appendix 1. MAINTAIN if-then-because statements to outline initial programme theory.**

| **MAINTAIN if-then-because statements to illustrate initial programme theories.** | | | **Context** | **Mechanism resource** | **Mechanism response** | **Outcome** |
| --- | --- | --- | --- | --- | --- | --- |
| **1** | **If the workforce receives appropriate training and a supportive MDT environment** | **If** staff operate in a supportive organisational context that enables training and MDT collaboration, **then** they are more likely to develop the confidence and skills needed to deliver tailored, person-centred care, **because** the training equips them with practical knowledge to meet the needs of people with dementia and their carers. | Context 1 - Supportive organisational context | MResource 1- Workforce training | MResponse 1 – Increased staff confidence  MResponse 2 – Increased staff skills | Outcome 1 – Improved skills for delivering person-centred and tailored care |
| **2** | **If intervention sessions are delivered frequently at home** | **If** staff deliver frequent, in-home sessions in a context where participants require structure and support to initiate activity, **then** engagement and adherence will improve, **because** consistent contact fosters routine, reinforces relevance, and promotes a sense of being supported. | Context 2 – People with dementia benefit from structure and in-home delivery | MResource 2 – Intervention frequently delivered at home | MResponse 3 - Increased motivation to engage in intervention activities  MResponse 5 – Individuals feel supported and valued  MResponse 7 - Reinforcement and routine-building | Outcome 2 – Increased independence with ADLs  Outcome 3 -Reduction in falls and injuries  Outcome 4 – Enhancement in QoL |
| **3** | **If home environment is improved for falls-safety** | **If** staff provide home modification advice and assist with physical changes in a context where participants have mobility challenges and face environmental fall risks, **then** participants will feel more confident and mobilise more safely, **because** the environment becomes safer and better supports their mobility needs. | Context 3 - Home setting  Context 4 - Mobility challenges | MResource 3 – Provide advice and assist with home modifications and fall prevention strategies | MResponse 3 - Increased motivation to engage in intervention activities  MResponse 4 – Increased confidence of PLwD and/or carer | Outcome 2 – Increased independence with ADLs  Outcome 3 -Reduction in falls and injuries  Outcome 4 – Enhancement in QoL |
| **4** | **If participants receive comprehensive care through a MDT structure** | **If** an MDT is involved in a context where participants have complex and varied needs, **then** they will receive holistic care and timely referrals, **because** the team’s diverse expertise enables comprehensive assessment and coordinated planning. | Context 5 - Complex, varied needs | MResource 4 – Holistic assessment and care through MDT  MResource 5 – Referrals for additional support | MResponse 5 – Individuals feel supported and listened to  MResponse 6 - Activation of external support networks to reduce social isolation | Outcome 4 – Enhancement in QoL |
| **5** | **If participants are assigned a dedicated care team** | **If** the participant’s care team are identified and engaged, **then** the participant is more likely to build trust, communicate openly, and feel motivated to engage, **because** consistent relationships enable person-centred care. | Context 6 - Need for continuity | MResource 6 – Dedicated care team for person-centred care | MResponse 3 - Increased motivation to engage in intervention activities  MResponse 4 – Increased confidence of PLwD and/or carer  MResponse 5 – Individuals feel supported and listened to | Outcome 2 – Increased independence with ADLs  Outcome 3 – Reduction in falls and injuries  Outcome 4 – Enhancement in QoL |
| **6** | **If the intervention is tailored** | **If** participants set their own goals in a context where individual needs, motivations, and cognitive abilities vary, **then** staff can tailor the intervention to those specific needs and abilities, **becaus**e personalising activities makes them more meaningful and achievable, which enhances engagement and adherence. | Context 5 - Complex, varied needs | MResource 7 – Tailored care | MResponse 3 - Increased motivation to engage in intervention activities  MResponse 5 – Individuals feel supported and listened to | Outcome 2 – Increased independence with ADLs  Outcome 3 – Reduction in falls and injuries  Outcome 4 – Enhancement in QoL |
| **7** | **If the intervention provides supportive tools** | **If** the intervention includes aids (e.g., cue cards, exercise breakdowns, handrails) in a context where participants have cognitive and physical impairments, **then** they will receive enhanced support, **because** these aids help them better understand and follow their activity plans, resulting in increased confidence and adherence. | Context 4 - Mobility challenges  Context 7 - Cognitive impairment | MResource 7 – Tailored care  MResource 8 – Supportive tools | MResponse 3 - Increased motivation to engage in intervention activities  MResponse 4 – Increased confidence of PLwD and/or carer  MResponse 5 – Individuals feel supported and listened to | Outcome 2 – Increased independence with ADLs  Outcome 3 – Reduction in falls and injuries  Outcome 4 – Enhancement in QoL |
| **8** | **If functional activities are embedded in everyday life** | **If** participants are provided with functional daily tasks embedded in everyday life in a context where routine and familiarity support learning and memory, **then** these activities can become more habitual over time, **because** they align with the participants’ existing routines, leading to increased independent task initiation by the person living with dementia and greater confidence from the carer in their ability to engage independently. | Context 2 – People with dementia benefit from structure and in-home delivery | MResource 9 – The MDT sets functional tasks that can be integrated into everyday life | MResponse 7 - Reinforcement and routine-building  MResponse 4 – Increased confidence of PLwD and/or carer | Outcome 2 – Increased independence with ADLs |
| **9** | **If knowledge is assessed and enhanced** | **If** knowledge of dementia and falls, including attitudes toward risk, is assessed in a context where participants hold varied beliefs and understanding about dementia and fall prevention, **then** staff will gain insights into participants' current understanding and attitudes, **because** this information enables targeted education to address knowledge gaps and promote more positive and proactive approaches to risk management. | Context 8 - Varied knowledge and beliefs | MResource 10 – Assessment and education by MDT | MResponse 8 - Develop risk management skills | Outcome 2 – Increased independence with ADLs |
| **10** | **If the carer supports the intervention** | **If** people living with dementia and their carers are enrolled as dyads in the intervention in a context where memory difficulties and reduced motivation are common, **then** the carer can provide prompts to encourage the completion of activities, **because** this support helps compensate for cognitive impairments and sustains engagement. | Context 7 - Cognitive impairment | MResource 11 - Carer support and prompting | MResponse 3 - Increased motivation to engage in intervention activities  MResponse 7 - Reinforcement and routine-building | Outcome 2 – Increased independence with ADLs  Outcome 3 – Reduction in falls and injuries  Outcome 4 – Enhancement in QoL |
| **11** | **If carer stress and burden is reduced** | **If** the intervention evaluates carer stress and provides guidance for additional support when necessary, in a context where carers often experience high levels of burden and emotional strain, **then** carers will be better prepared to oversee the intervention activities, **because** the additional support helps reduce stress and improves their overall well-being. | Context 9 - High carer burden | MResource 12 – Assessment and signposting by MDT | MResponse 4 – Increased confidence of PLwD and/or carer | Outcome 2 – Increased independence with ADLs  Outcome 3 – Reduction in falls and injuries  Outcome 4 – Enhancement in QoL |

**Appendix 2. The consolidated criteria for reporting qualitative research (COREQ) checklist for the pilot MAINTAIN process evaluation.**

| **Topic** | **Item No.** | **Guide Questions/Description** | **Reported on Page No.** |
| --- | --- | --- | --- |
| **Domain 1: Research team and reflexivity** | | | |
| *Personal characteristics* | | | |
| Interviewer/facilitator | 1 | Which author/s conducted the interview or focus group? | 5 |
| Credentials | 2 | What were the researcher’s credentials? E.g. PhD, MD | 5  Appendix 9 |
| Occupation | 3 | What was their occupation at the time of the study? | 5 |
| Gender | 4 | Was the researcher male or female? | Appendix 9 |
| Experience and training | 5 | What experience or training did the researcher have? | Appendix 9 |
| Relationship established | 6 | Was a relationship established prior to study commencement? | 5 |
| Participant knowledge of the interviewer | 7 | What did the participants know about the researcher? e.g. personal goals, reasons for doing the research | 5 |
| Interviewer characteristics | 8 | What characteristics were reported about the interviewer/facilitator? e.g. Bias, assumptions, reasons and interests in the research topic | Appendix 9 |
| **Domain 2: Study design** | | | |
| **Theoretical framework** | | | |
| Methodological orientation and theory | 9 | What methodological orientation was stated to underpin the study? e.g. grounded theory, discourse analysis, ethnography, phenomenology, content analysis | 2 |
| **Participant selection** | | | |
| Sampling | 10 | How were participants selected? e.g. purposive, convenience, consecutive, snowball | 5 |
| Method of approach | 11 | How were participants approached? e.g. face-to-face, telephone, mail, email | 5 |
| Sample size | 12 | How many participants were in the study? | 5  Appendix 4 |
| Non-participation | 13 | How many people refused to participate or dropped out? Reasons? | 5  Appendix 4 |
| **Setting** | | | |
| Setting of data collection | 14 | Where was the data collected? e.g. home, clinic, workplace | 5 |
| Presence of non-participants | 15 | Was anyone else present besides the participants and researchers? | Unknown |
| Description of sample | 16 | What are the important characteristics of the sample? e.g. demographic data, date | Appendix 4 |
| **Data collection** | | | |
| Interview guide | 17 | Were questions, prompts, and guides provided by the authors? Was it pilot tested? | Appendix 6 |
| Repeat interviews | 18 | Were repeat interviews carried out? If yes, how many? | Appendix 6 |
| Audio/visual recording | 19 | Did the research use audio or visual recording to collect the data? | 5 |
| Field notes | 20 | Were field notes made during and/or after the interview or focus group? | 5 |
| Duration | 21 | What was the duration of the interviews or focus group? | Appendix 6 |
| Data saturation | 22 | Was data saturation discussed? | N/A |
| Transcripts returned | 23 | Were transcripts returned to participants for comment and/or correction? | Appendix 6 |
| **Domain 3: Analysis and findings** | | | |
| **Data analysis** | | | |
| Number of data coders | 24 | How many data coders coded the data? | Appendix 7-9 |
| Description of the coding tree | 25 | Did authors provide a description of the coding tree? | Appendix 7-8 |
| Derivation of themes | 26 | Were themes identified in advance or derived from the data? | 5-6 |
| Software | 27 | What software, if applicable, was used to manage the data? | 6 |
| Participant checking | 28 | Did participants provide feedback on the findings? | Appendix 6 |
| **Reporting** | | | |
| Quotations presented | 29 | Were participant quotations presented to illustrate the themes/findings? Was each quotation identified? e.g. participant number | 8-14  Appendix 10 |
| Data and findings consistent | 30 | Was there consistency between the data presented and the findings? | Discussion |
| Clarity of major themes | 31 | Were major themes clearly presented in the findings? | 8-10 |
| Clarity of minor themes | 32 | Is there a description of diverse cases or discussion of minor themes? | 8-10 |

Developed from: Tong A, Sainsbury P, Craig J. Consolidated criteria for reporting qualitative research (COREQ): a 32-item checklist for interviews and focus groups. *International Journal for Quality in Health Care*. 2007. Volume 19, Number 6: pp. 349 – 357.

**Appendix 3. Site characteristics for the MAINTAIN pilot trial.**

**Site 1 – Intervention site**

Site 1 is embedded in an NHS Trust that provides a full range of mental health services, as well as support for people with learning disabilities and individuals with autism. The team delivering the intervention were an NIHR Research Delivery Team who are a member of the local Clinical Research Network (CRN). The team operates within the Medical Directorate, overseeing clinical research initiatives focused on mental health throughout the county. They administer Research Governance for ongoing studies, with several being affiliated with the NIHR but also supporting alternatively funded projects. At any given time, the team will concurrently manage multiple research projects, each at various stages of progress.

The team comprises a senior research nurse, three research nurses, and four clinical research practitioners. The MAINTAIN team consisted of a band 7 Parkinson's specialist physiotherapist, a band 7 occupational therapist (OT), and three rehabilitation support workers (RSW). One RSW was a band six mental health nurse and two RSWs were band four. Despite the clinical backgrounds of the team members, their research roles are distinct and independent. They do not have clinical responsibilities and must therefore collaborate with clinicians or internal/external agencies for recruitment purposes. The local site Principal Investigator is from within the Trust and other team members have worked for the Trust for many years so they have established relationships with colleagues who may be able to recruit. They also have access to Join Dementia Research and Age UK (e.g. I Forget Service). Several referrals have come from Dementia Support Workers who are employed by the local Community Mental Health Team. One of the RSWs used to work in an NHS Memory Clinic and does a lot of volunteering with dementia cafes and this has boosted recruitment rates.

The team held weekly multidisciplinary meetings and formal supervision sessions as well as having a therapy support WhatsApp group.

**Site 2 – Intervention site**

Site 2 operates as an independent charity that facilitates research and offers assistance to individuals affected by dementia and other conditions associated with older age, as well as their families and caregivers. The clinical researcher and RSW were both Site 2 employees and were both research psychologists (also known as assistant psychologists in the NHS). Site 2 employees did not have their usual clinical duties reduced when they were running MAINTAIN. Site 2 employed the services of an OT and a physiotherapist from a small private practice offering assessment and treatments in people’s homes. The physiotherapist and OT would do a joint home assessment and then discuss the participant's goals and activity programme afterwards. The site did not hold an MDT with the RSW and no supervision was offered to the RSW. Participants were recruited by calling previous service users to determine eligibility and by referrals from clinicians working within the site (e.g. doctors from the memory service).

**Site 3 – Intervention site**

Site 3 was embedded within an NHS Foundation Trust that provides comprehensive NHS services throughout a person’s life, encompassing physical and mental health care, delivered in various settings including general practices, hospitals, the community, and patients' homes. The majority of services are delivered within the community, focusing on treating individuals in their homes or at nearby clinics. When community care is not feasible, the Trust offers a range of facilities for treatment in hospital or residential environments. Additionally, healthcare services are provided in prisons across the local area.

The Clinical Researcher was a Research Practitioner within the local NHS-based Research Office that serves a partnership of local NHS Trusts, primary care organisations, social care services, and various settings outside the NHS. As a research office, the team provides various services to support studies from initial concept to delivery. This includes processing sponsorship requests for staff employed by partner trusts and preparing the necessary review contracts for site participation. Dedicated teams support research in primary care and settings outside the NHS, coordinating study set-up, site selection, and delivery. The delivery teams assist trusts with engaging services, identifying patients, and obtaining informed consent. The research office has formal partnerships with several local NHS Trusts that provide mental health and community services. Collaboration also extends to various allied academic institutions and other organisations.

Participants were recruited through a Rapid Access Service, which encompassed the Urgent Community Response, Virtual Ward, and Discharge to Assess pathways. The Rapid Access Service offers three distinct pathways focused on admission avoidance and early discharge. The multidisciplinary team includes Advanced Nurse Practitioners, Paramedics, Occupational Therapists, Physiotherapists, Doctors, Nursing Associates, and Senior Healthcare Assistants. The primary aim of the service is to support individuals with immediate health or functional needs who might otherwise require hospitalisation. It is part of a broader range of intermediate care services providing therapy and rehabilitation in community settings for adults in the local community who have conditions such as urinary tract infections, chest infections, heart failure, dehydration, frailty, falls, and hypertension.

Screening of potential participants was conducted by the Virtual Ward clinical team, who then referred eligible individuals to the Clinical Researcher based at the local NHS Research Office. Despite these established processes, recruitment at this site proved challenging, with no participants ultimately enrolled. Subsequent interview data with the Clinical Researcher highlighted several potential contributing factors to these recruitment difficulties. These included the Clinical Researcher not being physically integrated within the recruiting team, the clinical acuity of the service users being incompatible with an intervention focused on functional maintenance, and the competing demands on clinicians' time, where clinical priorities necessarily took precedence over research activities.

**Site 4 – Treatment as usual site**

Site 4 is part of an NHS Foundation Trust that provides mental health, learning disabilities, and neuro-rehabilitation services. The team are part of the NIHR Portfolio Research Delivery Team that, in a typical year, hosts between 50 to 60 large-scale NIHR projects. As a vital component of the regional research infrastructure, the site serves as the host for the local NIHR Applied Research Collaboration.

The site maintains two dedicated research registers that allow interested participants to join a mailing list for updates about relevant research opportunities. Everyone who goes through the local Memory Clinic has the option to sign up for the register. The Clinical Researcher was a Mental Health Nurse and had no clinical duties.

**Site 5 – Treatment as usual site**

Site 5 is embedded within an NHS Trust that provides community and mental health services. The Trust collaborates with schools, local hospitals, GP practices, social services, and other local authority departments such as housing and education. Additionally, partnerships with voluntary organisations and local community groups are established to achieve goals and ensure that care recipients are treated to the highest possible standard. Care and support are delivered through three directorates, (i) mental health services, (ii) families, young people, children’s, learning disability and autism services, and (iii) community health services. The Trust supports a wide range of research activities. At any given time, it backs several NIHR CRN portfolio studies, with participants drawn from various service areas and staff groups. This research is facilitated by the CRN-funded Research Delivery Team and is authorised through the Trust's R&D Office.

The two MAINTAIN Clinical Researchers support the CRN dementia portfolio. Participants were referred through GP surgeries doing database searches on their behalf, and also, via the falls clinic, and also, via the mental health for older adults team, including the memory service. The Research Delivery Team operates independently, initiating contact with clinical teams and collaborating on research activities, while maintaining a distinct separation from the clinical services themselves. The CRN staff all have research-specific areas including dementia and the Clinical Researchers had several years of experience in research.

**Site 6 – Treatment and usual site**

Site 6 operates within one of England's largest NHS Foundation Trust teaching hospitals. This multi-hospital Trust provides comprehensive healthcare services to a demographically diverse population. The Trust maintains strong academic partnerships to facilitate clinical research, with research activities fully integrated within clinical services to support evidence-based practice implementation. Participant screening was conducted by a consultant from the Older People's team.

**Appendix 4. Description of the MAINTAIN pilot process evaluation sample.**

**Supplementary Table 1. The number of participants who consented, withdrew, and were interviewed for the MAINTAIN process evaluation.**

|  | **Number PLwD consented to an interview** | **Withdrawn from interview** | **Number still eligible for an interview** | **Number PLwD interviews conducted** | **Number carers consented to an interview** | **Withdrawn** | **Number still eligible for an interview** | **Number carer interviews conducted** |
| --- | --- | --- | --- | --- | --- | --- | --- | --- |
| **Site 1** | 0 | 0 | 0 | 0 | 0 | 0 | 0 | 0 |
| **Site 2** | 4 | 2 | 2 | 1 | 7 | 3 | 4 | 3 |
| **Site 3** | 7 | 1 | 6 | 2 | 8 | 0 | 8 | 6 |
| **Total** | **11** | **3** | **8** | **5^±^ (62.5%)** | **15** | **4** | **11** | **9^*^ (82%)** |

±3 participants who consented to an interview were unable to engage in the process

*2 participants have been contacted several times with no response

**Abbreviations:** PLwD – People living with dementia

**Note:** Of the 18 participants recruited into MAINTAIN, 11 initially consented to the process evaluation. There were no significant demographic differences between those who consented and those who declined (n = 7). Reasons for declining the process evaluation were not collected; only reasons for main trial non-participation were recorded and will be reported separately.

**Supplementary Table 2. Demographics of the healthcare staff and clinical researcher who took part in the process evaluation**

| **Healthcare staff descriptors and demographics** | |
| --- | --- |
| **Roles on MAINTAIN**  Clinical Researcher:  Rehabilitation support worker:  Physiotherapist:  Occupational therapist:  Service manager: | 6  3  2  2  1 |
| Mean age (range) | 41.6 (23-62) |
| Gender | Female: 100% |
| **Highest education level**  PhD:  MSc:  Advanced Diploma:  Degree:  BSc:  Diploma: | 1  5  1  1  3  1 |
| **Ethnicity**  White:  Mixed (White other & Black African): | 12  1 |
| **Previous dementia experience**  Yes:  No: | 13  1 |
| **Clinical Duties Alongside MAINTAIN**  Yes:  No: | 8  6 |
| **Other Research Activities Outside of MAINTAIN**  Yes:  No: | 9  5 |

**Note:** One clinical researcher and two therapists were contacted on two occasions during the trial to invite participation in an interview; however, no response was received.

**Supplementary Table 3. Demographics of individuals living with dementia and caregivers who took part in the process evaluation.**

| **Demographics** | **Person living with dementia (n=5)** | **Caregivers (n=9)** |
| --- | --- | --- |
| **Mean age (range)** | 76 (61-93) | 69.6 (47-81) |
| **Gender**  **Female:**  **Male:** | 4  1 | 4  5 |
| **Number of participants providing informed consent** | 100% | 100% |
| **Ethnicity**  **White:** | 5 | 9 |
| **Mean Mini-ACE (range)** | 18.2 (15-24) |  |
| **Living arrangements** | Lives with spouse/partner: 4  Lives alone: 1 |  |

**Note:** There were two missing mini-ACE scores.

**Appendix 5. Fidelity checklists.**

1.
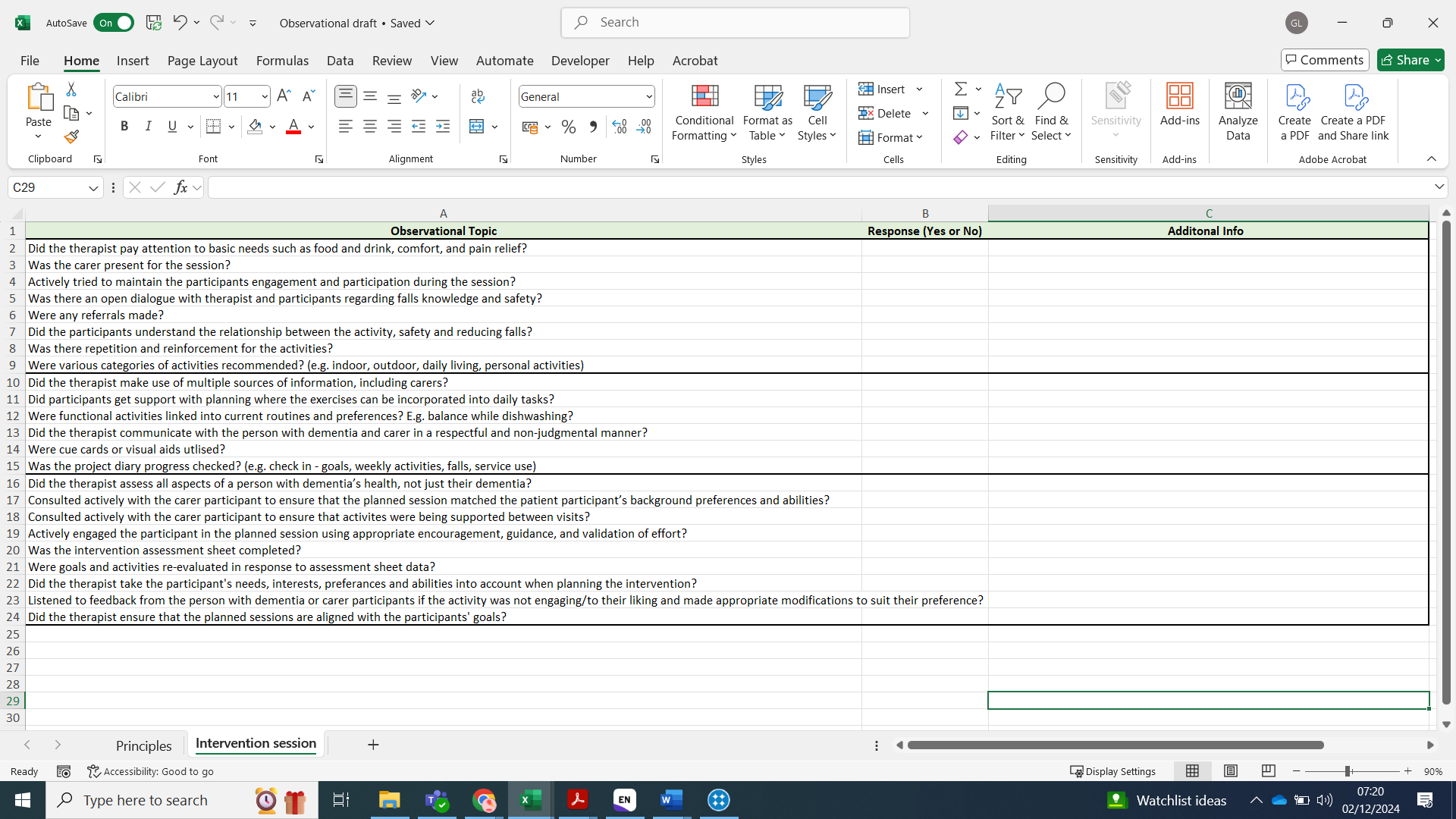
**MAINTAIN therapy sessions.**
2. **MAINTAIN multidisciplinary team meetings.**


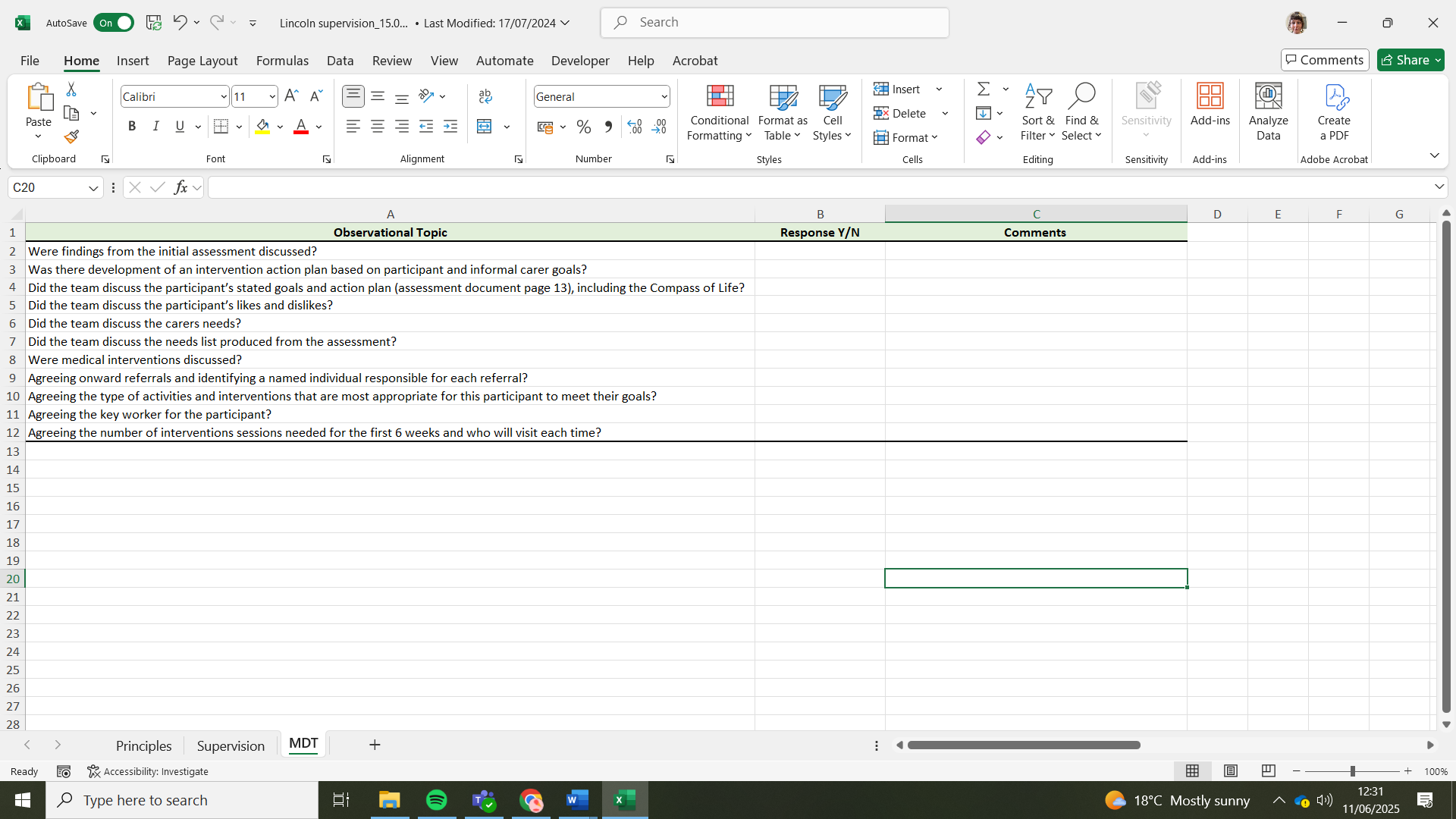


1. **MAINTAIN supervision.**


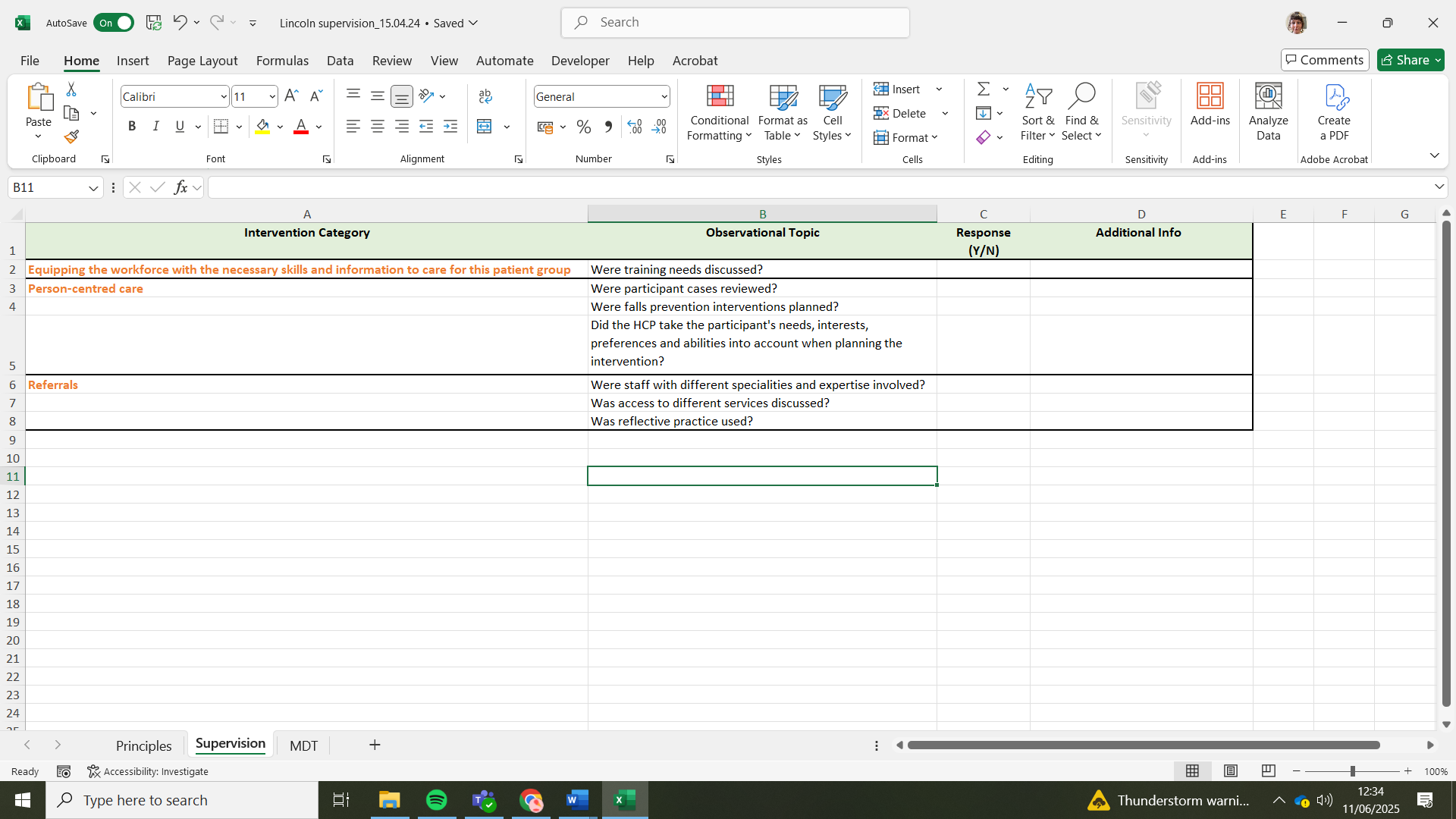


**Note on Scoring Approach:** Fidelity checklists were completed by LG (the observing researcher). If a core component of the MAINTAIN intervention, such as multidisciplinary team involvement, person-centred care, or caregiver engagement, was observed during a session, it was recorded as ‘yes’. This simple binary approach was considered appropriate for a pilot feasibility study, where the primary aim was to assess general adherence rather than quantify intensity or frequency.

**Appendix 6. Interview guides for the Maintain pilot trial.**

**This guide was not pilot-tested due to the small sample size of the pilot study.**

**Interview guide for people living with dementia**

1. Can you tell me why you decided to take part in this research?

- (Prompt: Differentiate between intervention and trial. Prompt for any reservations they might have had, and whether experiences matched up to their expectations at the start)
- Context: What factors influenced your decision to participate?

1. This study randomly put people in two groups – one that got the intervention sessions and one that didn't. When they told you about MAINTAIN, do you remember what you felt about the chance of not being in the group that got the program?

- Mechanism: How did this random assignment affect your motivation to take part?

1. What was your experience of completing the questionnaires?

- (Prompt: do you think there were too many or not enough?
- Mechanism: How did this impact your engagement?

1. The MAINTAIN team visited your home to check various things like how you move around, if there are any potential risks for falls, and to make sure you're physically doing well. What were your thoughts about this visit?

- (Prompt: Did you make/keep the suggested changes? Were the changes effective to reducing falls?)

**Staff**

1. Can you describe the MAINTAIN sessions in terms of who visited and what you did during those visits?

- (Prompt for setting of goals, content of sessions, what type of professional(s) visited).
- How did the interaction with staff affect your engagement and outcomes? Was it important to have a key worker?

1. Could you tell me a bit about the staff delivering the intervention?

-Were they knowledgeable?

-How well did you think they communicated and interacted with you?

Mechanism: What aspects of staff interaction were most influential in your experience?

**Materials**

1. Were you given any activities/homework to do in between sessions, and if so, did you do them? (Prompt for reasons did (not) do them, and any impact)

Outcome: How did these activities influence your progress?

1. Did you receive any printed materials as part of the programme? (Prompt for whether used and if they supported or hindered engagement?)

**Tailored care/goals**

1. Do you feel the sessions were personalised or tailored for you? (Prompt for individualised goals)
2. How did the intervention fit into your daily life and routine?
3. Was setting goals useful for you or not?

‐ How did you choose goals? (Check if one or both participant/carer were involved)

‐ How much progress have you made?

‐ Do you feel you have as much help as you need to help you achieve your goals or would you have liked more?

-Was goal setting 50/50 or did one of you take the lead?

Mechanism: How did goal setting drive changes in behaviour or outcomes?

**Impact/Outcomes**

1. Thinking about the different elements of the sessions (e.g. physio exercises), what happened as a result of doing each of these things? (Go through elements individually. Prompt for impact ie. Causal link between programme elements and psychological/behavioural/health changes in participant and carer)

Outcome: Identify causal links between program elements and changes observed.

1. Can you describe any changes, positive or negative, that came about because of the intervention? (Prompt: Ask specifically about confidence and independence).
2. Would you say you enjoyed the sessions or not? What aspects did you like or not like?

**Logistics**

1. Do you think the individual sessions were too long or short?

-Did the overall programme last for the right amount of time or should it be more/fewer weeks long?

1. Were there any barriers or facilitators that influenced your ability to adhere to the intervention? (Prompt for contextual factors such as wider family support, medical conditions, living arrangements)

**Referrals**

1. Did you receive any referrals to other services as part of the programme, and if so was this helpful or not? (Prompt for which service/professional was referred to and why, include geriatrician referrals)

**Reach**

1. Who do you think would benefit most or least from this type of intervention?

Mechanism: What characteristics make an individual more or less likely to benefit from the intervention?

**Topic guide for therapist interviews**

**This guide was not pilot-tested.**

**Intervention Questions**

1. Can you describe your service and your role in the MAINTAIN intervention?
2. Can you tell me how the MAINTAIN intervention fits within your current job role? (prompt did you have a caseload to reduce, if so, how was this done?)
3. What training and/or supervision did you receive/provide for MAINTAIN (if any)? (Prompt for how this supported them or not, and whether would change anything such as additional training topics, whether had any previous training and how this impacted care)
4. Do you get any training or skills development via MDT? If so what?
5. Did you feel the training increased your confidence in working with people with dementia or your ability to deliver tailored care?
6. The MAINTAIN team usually includes geriatricians, physiotherapists, occupational therapists and support workers. From your perspective, what are the advantages and disadvantages of using professionals with this skill mix for MAINTAIN? (Prompt for value of geriatrician referrals and their impact on patients)
7. How do you coordinate your work in MAINTAIN with other members of the team, and with other professionals in different teams? (Prompt for MDT meetings and coordination arrangements e.g. for referrals)
8. How easy or difficult is it to deliver the intervention as described in the manual? (Prompt for whether been able (1) deliver each component of the intervention (2) deliver for the allotted time and frequency (3) contextual factors)

-Home assessment and therapy sessions

1. Have you been able to tailor the intervention and if so how? What effect do you think this has had?
2. Did you refer many people to other services? If so, how did you have knowledge about the other services – did training cover this? Already know about it and if so how? If someone was new how would they find out about it?
3. Have there been any challenges with tailoring? (Prompt for setting of individualised goals)
4. Have you observed any indications or had any feedback about the acceptability of the intervention for participants and carers?
5. Do you think the intervention has created any changes for the participant or carer, and if so how? (Prompt for specificity about different elements of the intervention and their impact e.g. on falls, ADL, mobility, fear of falling, quality of life)
6. Have you observed any factors which impact on the participant or carer’s ability to benefit from MAINTAIN (Prompt for contextual factors such as wider family support, medical conditions, communication issues, living arrangements)
7. Do you think any changes should be made to the intervention, and if so what? (Prompt for changes to target group, intervention components, training/supervision, materials such as manual)

**Topic guides for clinical research interviews**

**This guide was not pilot-tested.**

1. Could you please give a brief summary of your role and the service where you are employed? This will help me gain a clearer understanding of the participants being recruited through your service.
2. Was screening part of your job role? Can you describe your experiences with the screening process? (i.e. was it easy?)
3. What was your experience with recruitment?
4. Were there any specific challenges in approaching the target population?

-Getting in touch with participants

-Explaining the study (including randomisation)

-Sending PIS

-Gaining consent

1. What were the reasons for non-consent to participate?
2. What was your experience with data collection?
3. How practical and feasible do you think data collection processes were (questionnaires, interviews)? Would you change anything?
4. Do you think the research burden of questionnaires and interviews for participants is too much, too little or about right? (Prompt for reasons why)
5. Was there any political or organisational issues that affected you recruiting?

**Appendix 7. Examples of the realist analysis method as described by Jackson and Kolla (2012) to derive Context - Mechanism Resource - Mechanism Response - Outcome configurations (CMMOCs) from the Maintain data.**

**The criteria for identifying contexts, mechanism resources, mechanism responses, and outcomes.**

| **Concept** | **Definition** |
| --- | --- |
| Context | Conditions or factors that existed before the introduction of the intervention. This could include characteristics of the home environment, dynamics of relationships, or experiences of the interviewees. |
| Mechanism resources | Intervention activities or actions implemented as part of the intervention. These were designed to trigger specific processes or changes in the individuals or systems involved. These can be found in Table 1. |
| Mechanism responses | Behavioural, psychological, or social changes triggered by the intervention. |
| Outcome | A result or effect of the intervention that improved participants' health or general wellbeing. |

Context-Mechanism Resource-Mechanism Response-Outcome configurations (CMMOc) were coded directly from narratives (detailed in Appendix 7), generating 18 context codes, nine mechanism-resource codes, 11 mechanism-response codes, 10 outcome codes, and 103 total CMMOCs.

Example one

The below extract was allocated the coding sting of ‘MResponse3-MResource2-O2-O5’.

“So, you know, like I said, you know, obviously, it raised his spirits, and to have something to look forward to. Like, you know, oh, they’re going to come around **(MResponse 3- Motivation)**. You know? Like someone’s going to come and visit him **(MResoruce 2 - Frequent home visits)**, and he’s, you know, going to... so, that’s good for him as well, like that social interaction. But also, you know, like I didn’t realise how bad he was when they came physically. But one of his goals was to be able to stand up like, say, from like sitting position, just to stand up, and he couldn’t do that at the beginning. Well, when they had the review a month or so ago, he was able to do that **(Outcome 2 - Strength and balance to mobilise)**. So, you know? So, yeah, we’ve seen both the, you know, physical side, but also, the... even just the mental side **(Outcome 5 - Enhanced QoL)** of, you know, knowing that someone’s going to come around and going help him get... get him out of the house, you know, and to... to like signpost him as well to other places that he can go just to get out of the house.” (Carer)

Example two

The below extract was allocated the coding string of ‘MResource9-MResponse7-MResponse3-C1’.

*“Having a carer that’s heavily involved* ***(MResource 9 – Carer support and prompting)*** *is really, really, really beneficial, because they’re kind of… or like, a study partner. They’re kind of… they’re establishing that routine* ***(MResponse 7 – Reinforcement and routine building)****, we’ve got to do these activities, kind of pushing people* ***(MResponse 3- Increased motivation to engage in intervention activities)****, particularly… particularly if they’ve got apathy and low motivation or just, I don’t feel like doing it* ***(Context 1 – PLwD finds it hard to engage).*** *So, having somebody that’s quite healthy, quite fit, engage, really, really does help and they can almost push the participant.” (Therapist)*

Jackson, S. F., & Kolla, G. (2012). A New Realistic Evaluation Analysis Method: Linked Coding of Context, Mechanism, and Outcome Relationships. *American Journal of Evaluation*, *33*(3), 339-349. <https://doi.org/10.1177/1098214012440030>

**Appendix 8. The identification process for common and unique Context - Mechanism Resource - Mechanism Response- Outcome configurations (CMMOCs).**

Similar to other realist evaluation approaches [1], CMMOCs were grouped by mechanism resources (core intervention components) to examine how components triggered responses and outcomes across contexts. This systematic analysis process revealed patterns in causal relationships, identifying areas needing modification for a future trial [2]. Programme theory refinement was based on an iterative process involving team discussions and consultation with our PPIE group (see Appendix 9 for further details on the analytic and theory refinement).

Reflexive practices were employed throughout the analysis, examining how researcher positionality and interpersonal dynamics might influence findings. Analysis was conducted by authors LG and SMG before trial outcomes were known, and findings were reviewed with our patient and public involvement and engagement (PPIE) group to reflect on programme theory alignment.


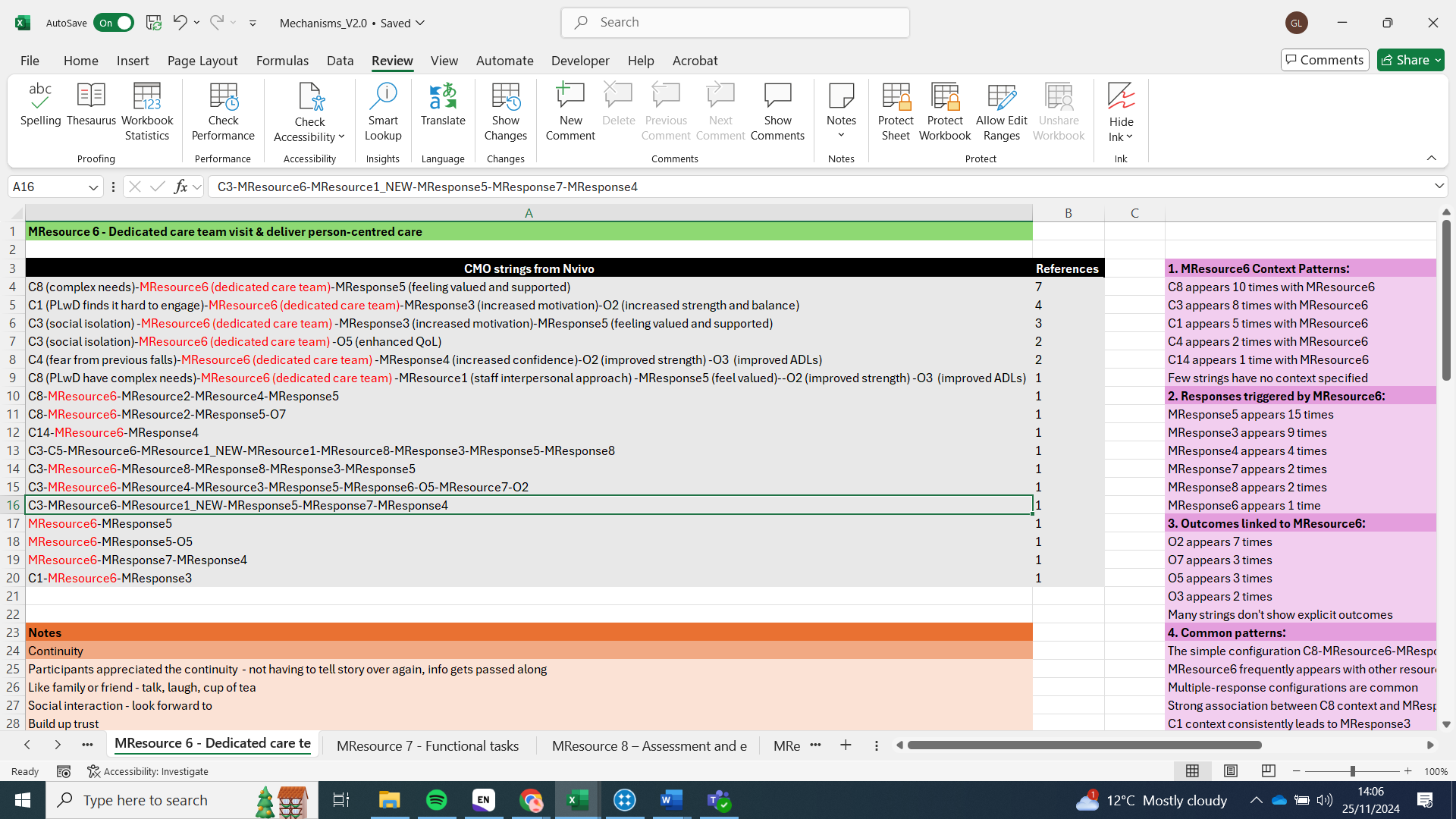


**Appendix 9: Expanded Description of Qualitative Data Analysis and Programme Theory Refinement**

This appendix outlines the process by which initial programme theories (IPTs) were developed, tested, and refined through qualitative analysis, in line with realist evaluation principles. It supplements the information provided in the manuscript’s qualitative analysis section.

**Development of Initial Programme Theories**

The IPTs were developed based on three core inputs:

1. Findings from the DIFRID feasibility study
2. Stakeholder consultations with people living with dementia, carers, and health/social care professionals
3. A review of relevant falls prevention literature in dementia care

These were synthesised into an initial logic model (Figure 1), structured around hypothesised causal pathways using ‘if–then’ propositions.

**Realist Analysis and CMMOC Development**

We employed a realist informed approach to examine how different aspects of intervention delivery activated mechanisms of change in various contexts and led to different outcomes. Interviews were anonymised, transcribed verbatim, and analysed using NVivo V.14.

Following Jackson and Kolla’s [26] approach, and using Dalkin’s [27] framing of mechanisms as comprising both resources and responses, transcripts were coded into Context (C), Mechanism–Resource (MR), Mechanism–Response (MRe), and Outcome (O) categories. This coding produced 103 Context–Mechanism–Mechanism–Outcome configurations (CMMOCs), covering:

- 18 context codes
- 9 mechanism–resource codes
- 11 mechanism–response codes
- 10 outcome codes

CMMOCs were grouped by core mechanism–resources (intervention components) to identify causal patterns across different settings and participants. This allowed systematic comparison of how similar resources triggered varied responses and outcomes depending on contextual conditions.

**Programme Theory Testing and Refinement**

We compared the resulting CMMOCs with the original IPTs to determine whether each was supported, refuted, or required refinement.

For instance:

- The IPT that signposting to other services would improve support access was refined, as participants, especially those with young-onset dementia, encountered structural barriers.

Patterns from these assessments informed the revised programme theory, represented in an updated logic model (Figure 2).

**Collaborative and Iterative Theory Refinement**

Programme theory refinement was a collaborative and iterative process. It involved:

- The core coding and thematic analysis were conducted by two researchers (LG and SMG), with regular input and discussion from the broader internal research team.
- Internal team meetings with the principal investigator, process evaluation research fellows, and allied health professionals delivering the intervention (physiotherapy, occupational therapy). These meetings examined CMMOC patterns against the IPTs, assessing explanatory coherence and clinical relevance.
- Two facilitated sessions with the Patient and Public Involvement and Engagement (PPIE) group. PPIE members reviewed original ‘if–then’ statements and reflected on whether refinements were needed.
- Reflexive analysis, in which researchers (LG and SMG) considered how positionality and interpersonal dynamics may have shaped interpretations. All analysis was conducted prior to unblinding of trial outcomes.

This process ensured that programme theories were not only data-driven but also grounded in stakeholder experience and practice insight. The revised theories will inform optimisation of the intervention for future trial testing.

**Note:** We note that the core analytic framework did not change during the evaluation. While our interpretations developed iteratively as familiarity with the data increased—consistent with realist and qualitative traditions—the coding structure, realist logic, and analytic procedures remained consistent throughout.

**Appendix 10. Additional results from the MAINTAIN analysis.**

**Frequent face-to-face home intervention delivery**

In our programme theory, we proposed that regular home visits would increase participant engagement through structure, support and real-world relevance. Our findings supported this theory, as the weekly home visits triggered increased motivation to engage in intervention activities:

*‘me coming every week is almost 'oh gosh, she's coming, we need to do this stuff and get on with it.’* (Therapist)

Caregivers particularly valued the structured visits, which provided meaningful interactions distinct from family contact. This proved valuable for participants with young-onset dementia, who often struggled with disrupted routines and loss of purpose:

‘*we're not retirement age, but I think, you know, we have to live the life of retirees. So, there's no structure in our day.’* (Caregiver)

Geographical disparities in service delivery emerged, with staff capacity constraints limiting access for participants in remote areas. Additionally, there were concerns about dependency on the intervention support, with therapists observing that without weekly visits, activity engagement might decline, particularly in contexts where caregivers faced high levels of responsibilities or health challenges:

*‘But if there’s somebody not coming in every week, I do wonder after the twelve-week period how often will those activities be done, particularly if a carer is having difficulty already establishing that routine.’* (Therapist)

**Holistic assessment and care**

The theory that therapists addressing multiple needs, including occupational needs, would help participants feel supported in achieving their goals and improving their quality of life was validated:

*‘The exercises have made my back and legs stronger because I can now stand in the bathroom and have a quick wash…Where before I couldn't do that…I think it's given me more confidence… And the exercise for my hands and fingers got me knitting again, ’* (Person with dementia)

Holistic assessment and care frequently worked well in contexts where participants had complex care needs, such as comorbidities:

*‘I've got so many damned things wrong with me. And if you do this, it impinges on that. .* (Person with dementia)

To support coordinated care and ensure a wider range of patient needs was addressed, therapists connected participants with additional services through referrals:

*‘I've been making onward referrals to other health professionals. That can be GPs, it can be podiatry…I did have a safeguarding issue.’* (Therapist)

Referral pathway effectiveness varied between sites, with one benefiting from direct NHS pathways while another struggled with uncertainty. Signposting was not always successful when referrals to complementary services revealed financial and age-related barriers, particularly affecting those with young-onset dementia:

*‘befrienders…they were too expensive. £18 an hour to come and have a cup of tea with you.’* (Person with dementia)

**Consistent person-centred care**

Our programme theory proposed that a consistent care team would build trust and engagement through person-centred relationships. This was supported by the findings: regular visits from the same team helped participants feel valued and supported, which in turn enhanced their motivation to engage.

*‘They have talked to her as she would like to be talked to…But above all, they’re very interested….’* (Caregiver)

Caregivers’ encouragement could cause tension, while therapists, trusted professionals outside the family, were often more effective. Consistent input from a dedicated team was crucial, given the typically fragmented care experienced by people with dementia.

*‘Before Sandra [therapist] and them come round, I didn't think I'd ever get any independence… people who'd been before…we never saw them again, never heard from them…Sandra [person living with dementia] and them did come back to you.’* (Caregiver)

**Functional tasks embedded in everyday life**

A core element of MAINTAIN was embedding functional tasks into daily life to build habits and support confidence and independence. Participants described three types of independence loss: the person with dementia’s declining ability, caregivers restricting activity due to safety concerns, and caregivers losing free time as responsibilities grew.

*‘when she was going to make a cup of tea, lifting the milk jug a few times…because she had really poor strength in her arms… building up her hand strength so she could carry it through and be able to pick up the kettle.’* (Therapist)

For participants with reduced physical capacity, therapists found they could not begin functional activities straight away. Instead, they started with basic physiotherapy exercises to build strength, gradually progressing towards functional tasks:

*‘They're all quite frail, and for us to be able to do functional tasks quite often to start with, we need to build up .’* (Therapist)

**Tailored care and goal-setting**

Our programme theory postulated that tailored care with individualised goals would make activities more meaningful and achievable. There was evidence that this was supported:

*‘I told them about strength in her legs, standing, and wanting, you know, the help, because it would help me… things are tailored round that*’ (Caregiver)

However, complex care needs and dementia symptoms—especially lack of insight—affected the effectiveness of tailored care. Caregivers felt therapists needed to understand both the dyad’s dynamics and dementia symptoms to set meaningful, achievable goals. One carer perceived a lack of such tailoring:

‘*It [tailored goal setting] shows a sort of lack of understanding of where that particular patient is…there are different things for each person...I think they were finding us quite difficult beside the requirements of the achievable goals.’* (Caregiver)

Therapists who were more experienced in dementia care showed a greater ability to adapt and tailor their therapy sessions to meet individual needs:

*‘So she'd come in with a plan, and then sometimes they'd deviate from the plan and kind of do it a different way that kind of fitted in with Steve.’* (Caregiver)

In comparison, therapists who specialised in general rehabilitation tended to focus on a more goal-orientated approach:

‘*I think she knew her programme of exercises…and whether Bex wanted her to push her knee or not, she was going to push it, because that was the programme of exercises.’* (Carer)

In terms of setting tailored goals, all therapists reported that the GAS was time-consuming, evidenced by its low completion rate in the fidelity data, as it often required post-home assessment session completion. Therapists also felt that the GAS was not conducive to establishing functional goals in dementia care contexts:

*‘ in this clientele, I think the cognitive ability needs to be taken into account when they're setting goals, because it's really hard to get them to come up with a realistic goal in the first place. And that includes the carer’* (Therapist)

Several participants felt they achieved the goals they set during MAINTAIN and they felt more confident and independent.

*‘And I wanted to be more independent, I really did, and I've gained that independence. And I wanted to be more confident, and, to a certain extent, I've got that confidence. So, I feel that MAINTAIN has done a great deal for me.’* (Person with dementia)
